# Supplementary material for: Comparison of Two Software Packages for Perfusion Imaging: Ischemic Core and Penumbra Estimation and Patient Triage in Acute Ischemic Stroke
Source: Cells. 2022 Aug 16;11(16):2547. doi: 10.3390/cells11162547 (PMC9406974; doi:10.3390/cells11162547)
Supplement: Supplementary file 1 [file cells-11-02547-s001.zip › cells-1751175-supplementary.pdf]

Supplementary Material

Table S1. Scan protocols of CTP.

Vender	SIEMENS	TOSHIBA
Manufacture's Model Name	SOMATOM Definition Flash	Aquilion ONE
Protocol Name	DynMulti 4D	Neuro ONE + mA Boost
Convolution Kernel	H31s	FC41
Brain Coverage	100 mm	160 mm
Field of View(FOV)	200 mm×200 mm	220 mm×220 mm
KVP	100	100
mAs	160	150
Matrix	512×512	512×512
Scanning Slice Thickness	5 mm	0.5 mm
Reconstruction Slice Thickness	5 mm	5 mm
Acquisition Time	60 s	53 s
Injection Rate	6 ml/s	6 ml/s
Onset	Immediately after injection	7 s after injection
Number of scanning phases	33	19

Table S2. Scan protocols of MRI.

**Vender**	**PHILIPS**	
**Manufacture's Model Name**	**Ingenia**	
**Field strength**	**3T**	
**Modality**	**DWI**	**PWI**
Sequence	Echo Planar Imaging(EPI)	Dynamic magnetic sensitivity enhanced MRI gradient EPI
Repetition Time(TR)	8 s	1618 s
Echo Time(TE)	96 ms	40 ms
Inversion Time(TI)	/	681 ms
Interslice gap	7.3 mm	4 mm
Slice thickness	6 mm	4 mm
Field of view(FOV)	228 mm×228 mm	224 mm×224 mm
Matrix	192×192	128×128
Number of Excitations(NEX)	2	2
Flip angle	90°	75°
b value	0, 1000	/
Injection Rate	/	5 ml/s
Onset	/	Immediately after injection
Number of scanning phases	/	60

Introduction S1

Stages of RealNow software processing

The stages of RealNow software processing can be summarized as: pre-processing, signal processing, perfusion and diffusion parameter calculation, lesion segmentation and mismatch calculation. The processing steps of these stages are described below.

1. Pre-processing

Medical imaging quality can affect digital imaging diagnosis. The analyzing process may corrupt because of ineligible image quality. The embedding algorithm of RealNow enhances data quality using pre-processing techniques.

1.1. Motion correction

To correct motions of time series data, registration is performed using a rigid-body registration with fourth-degree B-spline interpolation, and a least squares minimization. The average volume of the time series data serves as the reference image and each volume of the time series are registered to the reference image. Meanwhile, the similarity between each volume and the reference image is estimated and volumes with ineligible similarity are removed.

1.2. Spatial smoothing

Spatial smoothing is performed to reduce noise. Volumes at each time frame is filtered using the k-means clustering guided bilateral filter (KMGB)[1].

1.3. Brain extraction

Brain extraction is performed using a contour evolution technique[2]. Specifically, a slice with the largest brain section in axial views is located, and based on the location of this slice, contour evolution is performed for automatically extracting the brain in each slice. The segmented contour propagated from the previous slice is reused to penalize the defined object function for contour evolution. By doing so, the shape continuity between any two adjacent contours is enforced.

2. Signal processing

The signal intensity curves for each voxels represents the concentration of contrast agent for each of the tissue voxels and can be translated into concentration-time curves (CTC).

2.1. Estimation of the baseline signal

In order to convert the perfusion imaging signal into contrast concentration passing through the capillary bed, an estimation of the baseline perfusion imaging signal prior to the arrival of contrast agent is necessary. The pre-contrast baseline signal is calculated as the average signal over several time points after the establishment of a steady state.

2.2. Estimation of arterial input and Venous output function

RealNow selects locations for both arterial input (AIF) and venous output function (VOF). These parameters are optimized for robust delivery of AIF that estimates in anatomically appropriate locations under varying sequence parameters, slice prescription, acquisition, noise, and bolus shape.

Only a small fraction of the entire set of CTCs represents arteries. The majority of curves correspond to tissue voxels where the changes of signals are small. RealNow calculates the area under CTCs for each voxel and discarded sixty percent of the curves with the smallest area. Irregular and bumpy CTCs arise due to various scanning artifacts, and these curves with highly fluctuating time-courses are excluded. K-means cluster analysis (kCA)[3] is applied for partitioning the CTCs bundle into k groups. Since curves belonging to the same group exhibit common shape features, different groups can be distinguished.

RealNow chooses final AIF locations in a region with the highest sum of the spatially clustered values. We identify the height, h, arrival time, a, and width, w, of the CTCs signals at all spatial locations that represent brain tissue or vessels and computes their mean values $\bar{h}$, $\bar{a}$ and $\bar{w}$. Then, the AIF detection algorithm searches for spatially clustered locations with signals of above-average amplitude, below-average width, and early bolus-arrival time to identify possible locations for AIF. Specifically, the 'fitness' c of a voxel containing a suitable AIF is determined by the following cost function:

$$c = k_1(h - \bar{h}) + k_2(a - \bar{a}) + k3(w - \bar{w})$$

where the weighting coefficients $k_1 = 1.0, k_2 = -3.5, k_3 = -1.0$ are optimized empirically. The selection of the VOF employs a similar algorithm with the weighting coefficients $k_1 = 5.0, k_2 = 1, k_3 = -1.0$.

For the AIF selection, the search is limited to the anterior circulation, while for the VOF selection, only posterior locations are allowed.

3. Perfusion and diffusion parameters calculation

3.1. Deconvolution

Perfusion and other related hemodynamic parameters are estimated using deconvolution in the frequency domain. The mathematical method are as follows:

$$r(t) = \frac{1}{TR} FT^{-1}\{g(f)\frac{C_t(f)}{C_a(f)}\}$$

where

$$C_t(f) = FT\{c_t'(t)\}$$

and

$$C_a(f) = FT\{c_a'(t)\}$$

$c_t'(t)$ and $c_a'(t)$ are the $c_t(t)$ and $c_a(t)$ signals zero-padded to twice their length to avoid time-aliasing. TR is the sampling rate, $FT\{\}$ represents the Fourier transform, and f represents frequency, while $FT^{-1}\{\}$ is the inverse Fourier transform. Here, $g(f)$ is a regularization operator.

3.2. Perfusion parameters calculations

A number of useful hemodynamic parameters can be estimated from the deconvolved flow-scaled residue function. RealNow provides Cerebral Blood Volume (CBV), Cerebral Blood Flow (CBF), Mean Transit Time (MTT), and Time to the Peak of the Residue Function (T_{max}) maps.

$$\text{rCBV} = 100 \cdot \frac{k_{AV}}{\rho} \frac{1 - H_{SV}}{1 - H_{LV}} \frac{\int c_t(t)dt}{\int c_a(t)dt}, \quad [ml/100g]$$

$$\text{rCBF} = 100 \cdot 60 \cdot \frac{k_{AV}}{\rho}[max(r(t))], \quad [ml/100g/min]$$

$$\text{MTT} = \frac{60 \cdot \text{CBV}}{\text{CBF}}, \quad [s]$$

$$\text{T}_{\text{max}} = argmax[r(t)], \quad [s]$$

where $c_t(t)$ is perfusion image signal, $c_a(t)$ is arterial inflow function, $r(t)$ is tissue property, ρ is density of brain, H_{SV}, H_{LV} and k_{AV} are scaling factors.

3.3. Apparent diffusion coefficient calculations

For diffusion-weighted imaging (DWI) post-processing, the b=1000 images and b=0 images were applied, and apparent diffusion coefficient (ADC) is calculated as:

$$\text{ADC} = -\frac{1}{b} ln(\frac{b_{1000}}{b_0})$$

4. Lesion segmentation and Mismatch calculation

RealNow defines abnormal tissues with thresholds: ischemic core with a threshold as rCBF less than 30% for CTP or apparent diffusion coefficient (ADC) less than 620×10^{-6} mm^2/s for MRI, and penumbra with a threshold as T_{max} greater than 6 seconds. Notice that small objects with the volume below 1 ml and 3 ml are removed from the lesion mask after thresholding the CBF and T_{max} to eliminate the impact of noise. Finally, mismatch volume is calculated as the difference between penumbra volume and ischemic core volume, and mismatch ratio is the ratio of penumbra volume to ischemic core volume.

References

1. Pisana, F.; Henzler, T.; Schönberg, S.; Klotz, E.; Schmidt, B.; Kachelrieß, M. Noise reduction and functional maps image quality improvement in dynamic CT perfusion using a new k-means clustering guided bilateral filter (KMGB). *Med. Phys.* **2017**, *44*, 3464–3482.
2. Najm, M.A.; Kuang, H.; Fedorico; A. , Jogiat, U.; Goyal, M.; Hill, M.D.; Demchuk, A.; Menon, B.K.; Qiu, W. Automated brain extraction from head CT and CTA images using convex optimization with shape propagation. *Comput. Methods Programs Biomed.* **2019**, *176*, 1–8.
3. Mouridsen, K.; Christensen, S.; Gyldensted, L.; Ostergaard, L. Automatic selection of arterial input function using cluster analysis. *Magn. Reson. Med.* **2010**, *55*, 524–531.
